# Application of a Combination of a Knowledge-Based Algorithm and 2-Stage Screening to Hypothesis-Free Genomic Data on Irinotecan-Treated Patients for Identification of a Candidate Single Nucleotide Polymorphism Related to an Adverse Effect

**DOI:** 10.1371/journal.pone.0105160

**Published:** 2014-08-15

**Authors:** Hiro Takahashi, Kimie Sai, Yoshiro Saito, Nahoko Kaniwa, Yasuhiro Matsumura, Tetsuya Hamaguchi, Yasuhiro Shimada, Atsushi Ohtsu, Takayuki Yoshino, Toshihiko Doi, Haruhiro Okuda, Risa Ichinohe, Anna Takahashi, Ayano Doi, Yoko Odaka, Misuzu Okuyama, Nagahiro Saijo, Jun-ichi Sawada, Hiromi Sakamoto, Teruhiko Yoshida

**Affiliations:** 1 Graduate School of Horticulture, Chiba University, Matsudo, Chiba, Japan; 2 Plant Biology Research Center, Chubu University, Kasugai, Aichi, Japan; 3 Division of Genetics, National Cancer Center Research Institute, Tokyo, Japan; 4 Division of Medicinal Safety Science, National Institute of Health Sciences, Tokyo, Japan; 5 Division of Developmental Therapeutics, Research Center for Innovative Oncology, National Cancer Center Hospital East, Kashiwa, Chiba, Japan; 6 Gastrointestinal Medical Oncology Division, National Cancer Center Hospital, Tokyo, Japan; 7 Department of Gastrointestinal Oncology, National Cancer Center Hospital East, Kashiwa, Chiba, Japan; 8 Faculty of Horticulture, Chiba University, Matsudo, Chiba, Japan; 9 National Cancer Center Hospital East, Kashiwa, Chiba, Japan; 10 Division of Functional Biochemistry and Genomics, National Institute of Health Sciences, Tokyo, Japan; Geisel School of Medicine at Dartmouth College, United States of America

## Abstract

Interindividual variation in a drug response among patients is known to cause serious problems in medicine. Genomic information has been proposed as the basis for “personalized” health care. The genome-wide association study (GWAS) is a powerful technique for examining single nucleotide polymorphisms (SNPs) and their relationship with drug response variation; however, when using only GWAS, it often happens that no useful SNPs are identified due to multiple testing problems. Therefore, in a previous study, we proposed a combined method consisting of a knowledge-based algorithm, 2 stages of screening, and a permutation test for identifying SNPs. In the present study, we applied this method to a pharmacogenomics study where 109,365 SNPs were genotyped using Illumina Human-1 BeadChip in 168 cancer patients treated with irinotecan chemotherapy. We identified the SNP rs9351963 in potassium voltage-gated channel subfamily KQT member 5 (*KCNQ5*) as a candidate factor related to incidence of irinotecan-induced diarrhea. The *p* value for rs9351963 was 3.31×10^−5^ in Fisher's exact test and 0.0289 in the permutation test (when multiple testing problems were corrected). Additionally, rs9351963 was clearly superior to the clinical parameters and the model involving rs9351963 showed sensitivity of 77.8% and specificity of 57.6% in the evaluation by means of logistic regression. Recent studies showed that *KCNQ4* and *KCNQ5* genes encode members of the M channel expressed in gastrointestinal smooth muscle and suggested that these genes are associated with irritable bowel syndrome and similar peristalsis diseases. These results suggest that rs9351963 in *KCNQ5* is a possible predictive factor of incidence of diarrhea in cancer patients treated with irinotecan chemotherapy and for selecting chemotherapy regimens, such as irinotecan alone or a combination of irinotecan with a KCNQ5 opener. Nonetheless, clinical importance of rs9351963 should be further elucidated.

## Introduction

Genomic information has been proposed to be utilized as the basis for “personalized” health care. Interindividual variation in a drug response among patients has been well documented to cause serious problems in pharmacotherapy. This variation may be due to multiple factors such as disease phenotypes, genetic and clinical parameters (or environmental factors), and variability in the drug target or allergic response; all of these factors may affect both main and side effects [1], [2]. Although some biomarkers [3]–[9] have been proposed, it is still difficult to determine which group of patients will respond positively, which patients are nonresponders, and which may experience adverse reactions in cases where patients are administered the same medication dose. For effectiveness of personalized medicine in cancer chemotherapy, it is critically important to observe interindividual differences in a drug response and the role of genetic polymorphisms relevant to the drug metabolic pathways and drug response biology in pharmacogenomics [10].

**Figure 1:**
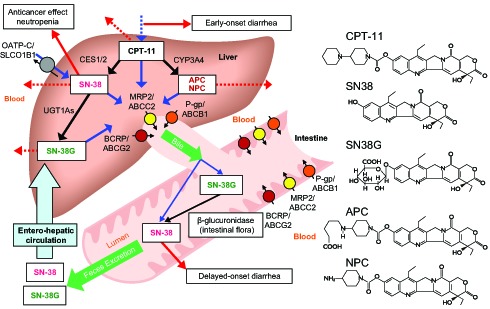
Drug metabolic pathways and the drug response of irinotecan.

Irinotecan (CPT-11), an anticancer prodrug, is widely used for the treatment of a broad range of carcinomas, such as colorectal, lung, ovarian, and cervical cancers. Unexpected severe diarrhea and neutropenia are prominent adverse effects of irinotecan treatment. The active metabolite SN-38 (7-ethyl-10-hydroxycamptothecin), a topoisomerase I inhibitor, is generated via hydrolysis of the parent compound by carboxylesterases [11], and is subsequently glucuronidated by uridine diphosphate glucuronosyltransferases (UGTs), such as UGT1A1, UGT1A7, or UGT1A9, to form an inactive metabolite, SN-38 glucuronide (SN-38G) [12]–[14]. Irinotecan is also inactivated by CYP3A4 to produce 7-ethyl-10- [4-N-(5-aminopentanoic acid)-1-piperidino] carbonyloxycamptothecin (APC; a major CYP3A4 product) and 7-ethyl-10-(4-amino-1-piperidino) carbonyloxycamptothecin (NPC; a minor product) [15], [16]. Irinotecan and its metabolites are excreted into the bile and urine via the action of ATP-binding cassette (ABC) transporters, such as P-glycoprotein (P-gp/ABCB1), multiple resistance-associated protein 2 (MRP2/ABCC2), and breast cancer resistance protein (BCRP/ABCG2) [17]. Transport of SN-38 from the plasma into the liver is mediated by the organic anion transporting polypeptide C (OATP-C/SLCO1B1) [18]. Most of the previous pharmacogenetic studies of irinotecan have been focused on *UGT1A1* polymorphisms and have shown clinical relevance of UGT1A1*28, a repeat polymorphism in the TATA box [-54_-39A(TA)_6_TAA>A(TA)_7_TAA or -40_-39ins TA], to severe adverse effects [3], [19], [20]. Based on these findings, in 2005, the Food and Drug Administration (FDA) of the United States approved an amendment for the formulation called Camptosar (irinotecan-HCl) (NDA 20-571/S-024/S-027/S-028) and for clinical use of a genetic diagnostic kit for the *28 allele. In parallel with this advance in the USA, clinical relevance to severe neutropenia of UGT1A1*6 [211G>A(G71R)], another low-activity allele detected specifically in East Asians, as well as *28, was demonstrated in several studies on Asian patients [5], [21]–[23]. Accordingly, in June 2008, the Ministry of Health, Labor, and Welfare of Japan approved changes to irinotecan formulations (Campto and Topotecin) by adding a warning about the risk of severe adverse effects in patients either homozygous or compound-heterozygous for UGT1A1*28 and *6 (*28/*28, *6/*6, *28/*6) and also approved clinical use of a diagnostic kit for UGT1A1*28 and *6. Severe adverse effects, however, are reported in patients without the genetic variations *6/*6, *28/*28, and *28/*6; therefore, several clinical studies have suggested that polymorphisms of the drug transporter genes, such as *ABCB1*, *ABCC2*, *ABCG2*, and *SLCO1B1*, might affect irinotecan pharmacokinetics (PK)/pharmacodynamics (PD) in Caucasian and Asian patients [22], [24]–[35], as shown in Fig. 1. Nonetheless, the almost all reported results deal with PK in patients and neutropenia induced by irinotecan as an adverse reaction not but with diarrhea. Therefore, other factors responsible for other irinotecan adverse effects, such as diarrhea should be identified.

Diarrhea induced by irinotecan is classified into early- and delayed-onset diarrhea, occurring within 24 hr or ≤24 hr after irinotecan administration, respectively [36]. Irinotecan induces early-onset diarrhea as one of adverse cholinergic effects (acetylcholinelike effects) by inhibiting acetylcholinesterase (AChE) and binding to muscarinic acetylcholine receptors (mAChR) [37], [38]. These inhibitory actions are induced by irinotecan, which has an amino group at the C-10 position [37]. Other than that, irinotecan induces delayed-onset diarrhea via rapid deconjugation of SN38G and adsorption of released free SN-38 by β-glucuronidase of intestinal flora [39]–[41], as shown in Fig. 1. In the present study, we focused on polymorphisms of genes with transporter activity to identify predictive factors of diarrhea induced by irinotecan because there are many genes related to transporter activity in both pathways.

A genome-wide association study (GWAS), also known as a whole-genome association study (WGA study, or WGAS), is an examination of many common genetic variants in different individuals to determine whether a particular variant is associated with a trait. GWAS using hypothesis-free genomic data is a powerful technique for identifying interindividual variation among patients. On the other hand, multiple testing problems are a limitation of this approach. To address this issue, we recently proposed a combined method consisting of a knowledge-based algorithm, 2 stages of screening, and a permutation test for identifying single nucleotide polymorphisms (SNPs) [7]. In general, the objective of a statistical or bioinformatic analysis is the enrichment of important information in a large dataset [42]–[47]. The use of a knowledge-based algorithm is not a novel concept, but is both practical and useful [48]–[66]. In the previous study, we found that rs2293347 in the gene of human epidermal growth factor receptor (*EGFR*) is a candidate SNP related to the chemotherapeutic response; we achieved this result by applying our combined method to gastric cancer patients who were treated with fluoropyrimidine [7]. However, our combined method was applied to only 1 dataset. Therefore, the usability of our combined method as a novel approach was still unclear.

We used the combined method in an actual genome-wide pharmacogenomics study of antitumor drugs, particularly irinotecan. We found that rs9351963 in the gene of potassium voltage-gated channel subfamily KQT member 5 (*KCNQ5*) is a candidate SNP related to the adverse response. Rs9351963 may be a potential predictive factor of incidence of diarrhea in cancer patients treated with the cancer prodrug irinotecan.

## Materials and Methods

### Ethics statement

The study was conducted according to the principles expressed in the Declaration of Helsinki, and the ethics committees of the National Cancer Center and National Institute of Health Sciences, Japan, approved the study protocol. All patients provided written informed consent to participate.

### Preparation of hypothesis-free genomic data for cancer patients treated with irinotecan

This study was performed within the framework of the Millennium Genome Project in Japan, and 4 antitumor drugs were chosen as project targets: gemcitabine, paclitaxel, fluoropyrimidine, and irinotecan. These drugs (alone or in some combination) were administered to approximately 1,000 cancer patients at the National Cancer Center, Japan. Additionally, approximately 1,000 DNA samples were extracted from peripheral blood mononuclear cells and 109,365 SNPs were genotyped using the Illumina Human-1 BeadChip. In this study, we focused on pharmacogenomic properties of irinotecan. Participants included 177 Japanese irinotecan-naïve cancer patients (56 cancer patients treated with irinotecan monotherapy and 121 cancer patients treated with irinotecan combination therapy) at the National Cancer Center Hospital and National Cancer Center Hospital East. A summary of the characteristics of the 176 patients is listed in Table S1. We excluded 1 patient who refused grading of adverse reactions. Furthermore, we excluded 8 patients who did not have genotyping data. Therefore, we analyzed the remaining 168 patients (53 cancer patients treated with irinotecan monotherapy and 115 cancer patients treated with irinotecan combination therapy) in the present study. We defined the 53 patients treated with irinotecan monotherapy as the first dataset and the 168 patients treated with irinotecan chemotherapy (consisting of irinotecan monotherapy and combination therapy) as the second dataset for 2 stages of screening.

### Monitoring and adverse effects

A complete medical history and data on physical examination were recorded before the irinotecan therapy. Complete blood cell counts with differentials and platelet counts, as well as blood biochemical variables, were measured once a week during the first 2 months of irinotecan treatment. Adverse events were graded according to the National Cancer Institute - Common Toxicity Criteria (NCI-CTC Version 2.0). Only the highest grade of adverse events was recorded during the first 2 months of irinotecan treatment for each patient and adverse event.

### Patient characteristics and clinical parameters

A summary of the patients' characteristics in the two datasets for diarrhea is shown in Table 1. The association of genetic or clinical parameters with incidence of grade ≥2 diarrhea was examined on the basis of Spearman's rank correlation coefficient. “UGT1A1*6 or *28” is an effective genetic predictive factor of irinotecan-induced neutropenia and pharmacokinetics in cancer patients [5]. This factor was constructed from 2 polymorphisms: UGT1A1*6 and *28.

**Table 1:** Irinotecan-treated cancer patients with SNP information, genetic factor, and clinical parameters for incidence of diarrhea.

Parameters		Diarrhea								
		Irinotecan monotherapy				Irinotecan chemotherapy (including monotherapy)				
		Number of patients		Spearman's rank correlation		Number of patients		Spearman's rank correlation		
		Grade <2	Grade ≥2	*ρ*	*p* value	Grade <2	Grade ≥2	*ρ*		*p* value
UGT1A1*6 or *28	0	15	5	0.056	6.89E–01	64	17	0.009		9.06E–01
	1	21	7			57	16			
	2	3	2			11	3			
Gender	Male	26	11	−0.114	4.15E–01	101	28	−0.012		8.75E–01
	Female	13	3			31	8			
Age		39	14	0.013	9.29E–01	132	36	0.080		3.02E–01
Area		39	14	0.010	9.45E–01	132	36	−0.054		4.88E–01
PS	<2	38	13	0.106	4.50E–01	130	35	0.039		6.15E–01
	≥2	1	1			2	1			
Smoking	0	37	14	−0.119	3.97E–01	111	30	0.008		9.13E–01
	1	2	0			21	6			
Alcohol	0	33	10	0.149	2.88E–01	90	26	−0.036		6.44E–01
	1	6	4			42	10			
Alb	0	18	10	−0.223	1.08E–01	71	24	−0.108		1.62E–01
	1	21	4			60	12			
	2	0	0			1	0			
Hg	0	14	4	0.061	6.65E–01	58	14	0.040		6.05E–01
	1	22	9			67	20			
	2	3	0			6	1			
	3	0	1			0	1			
	4	0	0			1	0			
GOT	0	33	12	−0.014	9.23E–01	108	32	−0.080		3.05E–01
	1	6	2			22	4			
	2	0	0			2	0			
ALP	0	28	8	0.117	4.05E–01	89	23	0.026		7.38E–01
	1	9	6			38	12			
	2	0	0			2	1			
	3	2	0			3	0			
Cr	0	31	13	−0.157	2.62E–01	124	35	−0.060		4.41E–01
	1	8	1			8	1			
C_max_/dose		39	14	0.049	7.31E–01	132	36	0.019		8.10E–01
AUC ratio		39	14	−0.078	5.81E–01	132	36	−0.109		1.60E–01
Concomitant drug - 5-FU	0	39	14	NA	NA	106	28	0.026		7.40E–01
	1	0	0			26	8			
Concomitant drug - CDDP	0	39	14	NA	NA	76	24	−0.076		3.28E–01
	1	0	0			56	12			
Concomitant drug - MMC	0	39	14	NA	NA	121	36	−0.138	†	7.40E–02
	1	0	0			11	0			
Concomitant drug - VP16	0	39	14	NA	NA	129	35	0.014		8.61E–01
	1	0	0			3	1			
Concomitant drug - Amrubicin	0	39	14	NA	NA	132	34	0.210	*	6.25E–03
	1	0	0			0	2			

“UGT1A1*6 or *28” is a genetic factor constructed from 2 polymorphisms (UGT1A1*6 and *28); “2” indicates *6/*6, *28/*28 or *6/*28, “1” indicates *6 or *28, and “0” indicates “other than 2 and 1.” Area: body surface area (m^2^), PS: performance status, Cr: grade of creatinine, Hg: grade of hemoglobin, Alb: grade of albumin, ALP: grade of alkaline phosphatase, and GOT: grade of glutamic oxaloacetic transaminase. Each laboratory test value (Alb, Hg, GOT, ALP, and Cr) was recorded before the irinotecan therapy. For each type of clinical tests the grade and aberrant values were defined according to the National Cancer Institute - Common Toxicity Criteria (NCI-CTC, Version 2.0). C_max_/dose: SN38 C_max_/dose [10^−3^×m^2^/L]. AUC: area under the concentration-time curve. AUC ratio: Ratio of AUC_SN38_/AUC_CPT-11_. 5-FU: 5-fluorouracil, CDDP: cisplatin, MMC: mitomycin C, VP16: etoposide. * and † indicate *p*<0.05 and 0.05≤*p*<0.10, respectively. For each concomitant drug, 0 means “not administered,” 1 indicates administered.

### Fisher's exact test

This statistical test is usually used to determine nonrandom associations between 2 categorical variables [67]. Fisher's exact test is similar to the chi-squared test. If a sample size is large, then the chi-squared test is suitable. Nevertheless, significance values from the chi-squared test are only approximated. Fisher's exact test is used in to analyze contingency tables when the sample sizes are small [67]. We used Fisher's exact test in the present study. The odds ratio (OR) is defined as *a*×*d*/(*b*×*c*), where *a* is the number of patients that had adverse events with a minor allele, *b* is the number of patients that did not have adverse events with a minor allele, *c* is the number of patients that had adverse events with a major allele, and *d* is the number of patients that did not have adverse events with a major allele. The null hypothesis for Fisher's exact test is OR = 1.

### The permutation test

The permutation test theory evolved from the works of Fisher and Pitman in the 1930s [68]. In this study, *p* values of multiple-comparison analyses were adjusted by applying the permutation test to 2 stages of screening. The case–control (or phenotype) labels were randomly shuffled for the 2 screening stages, and *p* values were calculated using Fisher's exact test. The lowest *p* value was selected for the randomized data. This procedure was repeated 100,000 times. Exact *p* values for the permutation test were calculated based on the distribution of the lowest *p* values.

### Multiple testing correction

The Bonferroni correction is a method used to address the problem of multiple comparisons (also known as the multiple testing problem). It is considered the simplest and most conservative method for control of the family-wise error rate (FWER). In addition, false discovery rate (FDR) controlling procedures, such as the Benjamini-Hochberg (BH) method [69], are more powerful (i.e., less conservative) than the FWER procedures, such as the Bonferroni correction, at the cost of increasing the likelihood of false positives within the rejected hypothesis. In the present study, the BH method was used to calculate the *q* value. The *q* value is defined as an FDR analog of the *p* value.

### The Akaike information criterion (AIC)

The AIC is a measure of the relative goodness of fit of a statistical model [70]. A smaller AIC indicates a better fit when comparing fitted objects. The AIC is defined according to the formula -2× (log likelihood) + (2×*n_par_*), where *n_par_* represents the number of parameters in the fitted model, and the log-likelihood value [71] is obtained from the logistic regression model.

### The receiver operating characteristic (ROC)

ROC analysis is a graphical plot that illustrates the performance of a binary classifier system as its discrimination threshold is varied. It is built by plotting sensitivity (the number of true positive results divided by the number of true positive samples) against (1 minus specificity) at various threshold settings. (Specificity is the number of true negative results divided by the number of true negative samples.) The area under the curve (AUC) of a ROC curve is an indicator of expected performance of the test. A higher AUC is more desirable, with a value of 1.00 denoting perfect performance (sensitivity and specificity are both 100%), while a value of 0.50 indicates random performance.

### Gene set based on gene ontology GO terms

GO has been developed to provide scientists with a controlled terminology system for labeling gene functions in a precise, reliable, computer-readable manner. Data for annotated genes and associated GO terms were obtained from the GO website (http://www.geneontology.org). We compiled a GO term list to select polymorphisms in genes with transporter activity (GO:0005215) and related activities, as shown in Table S2. The numbers of GO terms obtained was 943. GO data were obtained on July 1, 2010.

## Results

### Association analysis of adverse affects and clinical parameters (or a genetic factor)

The association between clinical parameters (or a genetic factor) and incidence of grade ≥2 diarrhea was examined on the basis of Spearman's rank correlation coefficient, as shown in Table 1. This table shows that no parameter was associated with the adverse response to chemotherapy (incidence of grade ≥2 diarrhea) in the first dataset (patients treated with irinotecan monotherapy). Nonetheless, Amrubicin (*p* = 0.00625) was significantly associated with the response in the second dataset (patients treated with any irinotecan chemotherapy: a combination or monotherapy). Mitomycin C (MMC; *p* = 0.0740) was weakly associated with the response. These clinical factors should be evaluated when constructing diagnostic models involving multiple factors.

### Extraction of candidate SNPs using the combined method consisting of the knowledge-based algorithm, 2 stages of screening, and the permutation test

**Figure 2:**
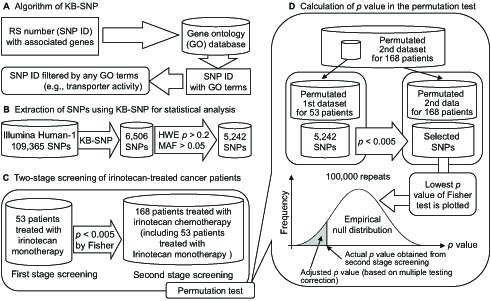
An outline of chemotherapeutic response analysis in irinotecan-treated cancer patients using a combined method consisting of a knowledge-based algorithm for identifying SNPs (KB-SNP), 2 stages of screening, and the permutation test. (A) The KB-SNP algorithm. (B) Extraction of SNPs using KB-SNP for statistical analysis. (C) Two-stage screening of irinotecan-treated cancer patients. (D) Calculation of the *p* value in the permutation test based on the 2 stages of screening. The second dataset (includes first dataset) was permutated. The permutated first dataset was extracted from the permutated second dataset. By using Fisher's exact test, SNPs with *p*<0.005 for the first dataset were selected from among 5,242 SNPs. Among the selected SNPs, those with the lowest *p* value in Fisher's exact test for the second dataset were selected. This procedure was repeated 100,000 times and empirical null distribution was constructed. Using the distribution, the actual *p* value obtained from the second stage of screening was converted to the adjusted *p* value (based on correction of multiple testing problems). At these screening steps, allele models were used for each SNP.

In this study, we applied the combined method to hypothesis-free genomic data on cancer patients treated with irinotecan chemotherapy as shown in Fig. 2. Figure 2A shows an outline of the knowledge-based algorithm for identifying SNPs (KB-SNP). In the previous study, we extracted rs numbers (SNP IDs) related to cancer using a combination of National Center for Biotechnology Information (NCBI) dbSNP and NCBI PubMed [7]. In the present study, we extracted rs numbers from genes linked to specific GO terms instead of the combination of NCBI dbSNP and PubMed. In this analysis, we defined specific GO terms as the terms related to transporter activity.

A total of 6,506 SNPs related to transporter activity were extracted from 109,365 SNPs using KB-SNP (Fig. 2B). Furthermore, we excluded SNPs with a *p* value <0.2 in the Hardy-Weinberg equilibrium (HWE) or the minor allele frequency (MAF) <0.05. Then the extracted 5,242 SNPs were used in the association study.

We analyzed 53 patients treated with irinotecan monotherapy as the first dataset for first-stage screening in the association study (Fig. 2C). Each *p* value was calculated using Fisher's exact test for the allele model. A total of 24 SNPs with *p*<0.005 were extracted. In the second stage of screening, 168 patients treated with irinotecan chemotherapy (including 53 patients treated with irinotecan monotherapy) were analyzed to validate these 24 SNPs. Adjustment of a calculated *p* value for the second stage of screening was conducted using the permutation test for these 2 stages of screening (Fig. 2D). Only rs9351963 in *KCNQ5* showed a statistically significant *p* value (0.0289), which was determined using the permutation test. The rs9351963 is a common variant (MAF = 0.328). Furthermore, we conducted Fisher's exact test and used the Benjamini-Hochberg method [69] to calculate *p* and *q* values for the second dataset only. Seven SNPs had a *q* value <1, as shown in Table 2. Six SNPs (rs11022922, rs3918305, rs3813627, rs768172, rs3813628, and rs10815019) had *q* = 0.802 as shown in Table 2. This result indicates that 5 out of 6 SNPs were false positive; however, we assessed performance of only rs9351963 in the process of model construction.

**Table 2:** Extracted 7 SNPs with *q*<1 for the second dataset.

RS number	Allele	MAF	SNP function		Chr	Position^a^	Associated gene symbol	For second dataset		Two stages of screening	
			Type	Location				*p*_F_	*q*_BH_		*p*_per_
rs9351963	A/C	0.328	cSNP	intron	6	73749861	*KCNQ5*	3.31E–05	0.173	*	0.0289
rs11022922	C/T	0.376	cSNP	intron	14	63472498	*KCNH5*	3.21E–04	0.802		1.0000
rs3918305	A/G	0.402	cSNP	intron	12	109331162	*SVOP*	6.21E–04	0.802		1.0000
rs3813627	G/T	0.435	cSNP	NearGene–5	1	161195148	*TOMM40L*	7.62E–04	0.802		1.0000
rs768172	A/T	0.441	cSNP	intron	7	95805703	*SLC25A13*	7.87E–04	0.802		1.0000
rs3813628	A/C	0.436	cSNP	5′UTR	1	161196166	*TOMM40L*	1.02E–03	0.802		1.0000
rs10815019	A/G	0.222	cSNP	intron	9	4547288	*SLC1A1*	1.20E–03	0.802		1.0000

RS number: reference SNP identification number in dbSNP, MAF: minor allele frequency, Chr: chromosome number, i.e., a position in human genome GRCh37.p10 build 104, *p*_F_ indicates a *p* value calculated using Fisher's exact test, *q*_BH_ indicates adjusted *p*_F_ value by the Benjamini-Hochberg method, *p*_per_ indicates *p* values adjusted using a permutation test for multiple testing problems, * indicates *p*_per_<0.05. NearGene-5 indicates that the SNP is within 2 kb upstream of a gene.

### Comparison of models based on rs9351963 in *KCNQ5*

**Figure 3:**
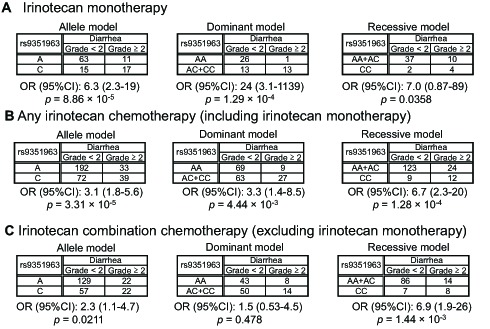
Contingency tables for rs9351963 in *KCNQ5* for each model using each dataset. (A) irinotecan monotherapy (first dataset), (B) any irinotecan chemotherapy (including irinotecan monotherapy; second dataset), and (C) irinotecan combination chemotherapy (excluding irinotecan monotherapy). OR: odds ratio. The *p* values were calculated using Fisher's exact test. CI: confidence interval.

We analyzed not only an allele model but also dominant and recessive models of rs9351963 in *KCNQ5* in relation to the first dataset (irinotecan monotherapy), the second dataset (any irinotecan chemotherapy), and the dataset of irinotecan combination chemotherapy (excluding irinotecan monotherapy), as shown in Figure 3. Figure 3A shows that the *p* value of the allele model was the lowest (*p* = 8.86×10^−5^, OR = 6.3), and the *p* value (*p* = 1.29×10^−4^, OR = 24) of the dominant model was lower than the *p* value (*p* = 0.0358, OR = 7.0) of the recessive model in the first dataset. In addition, Figure 3B shows that the *p* value of the allele model was the lowest (*p* = 3.31×10^−5^, OR = 3.1), and the *p* value (*p* = 1.28×10^−4^, OR = 6.7) of the recessive model was lower than the *p* value (*p* = 4.44×10^−3^, OR = 3.3) of the dominant model in the second dataset. Therefore, we evaluated the 3 models using the dataset of irinotecan combination chemotherapy (excluding irinotecan monotherapy; Fig. 3C). Figure 3C shows that the *p* value (*p* = 1.44×10^−3^, OR = 6.9) of the recessive model meant strong statistical significance and the OR was almost equal to OR ( = 7.0) in the first dataset, as shown in Figure 3A. Although ORs of the recessive models seemed to have high homogeneity among all 3 datasets, there was no statistical evidence. Therefore, the proportional odds model was used to construct multiple logistic regression models.

### Selection of the model of rs9351963 in *KCNQ5* and construction of multiple regression models

**Figure 4:**
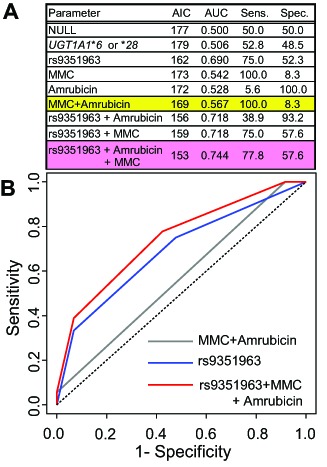
Comparison of AIC, AUC, and ROC curves for logistic regression models. (A) Parameters of each model. (B) The ROC curve of a model consisting of rs9351963+MMC+ Amrubicin. ROC: receiver operating characteristic, AUC: area under the ROC curve, NULL indicates the model without parameters. Each genetic factor conforms to the proportional odds model, AIC: Akaike's information criterion, AUC: area under the ROC curve, Sens.: Sensitivity (%), Spec.: Specificity (%).

We compared the AICs and AUCs using the second dataset in the 8 models: NULL (without parameter), “UGT1A1*6 or *28” (an integrated predictive factor based on polymorphisms related to neutropenia), and rs9351963 (genotype of rs9351963 in *KCNQ5*), Amrubicin, MMC, rs9351963+Amrubicin, rs9351963+MMC, and rs9351963+Amrubicin+MMC (Fig. 4A). Figure 4A shows that performance of all models except *UGT1A1* *6 or *28 is better than the performance of the NULL model. Although the Amrubicin+MMC (combination of Amrubicin and MMC) model was better than Amrubicin alone or MMC, the rs9351963 models were clearly better than the Amrubicin+MMC model, as shown in Figures 4A and 4B. Performance of rs9351963+Amrubicin and rs9351963+MMC models was better than performance of the rs9351963 model. Furthermore, performance of the rs9351963+Amrubicin+MMC model was better than that of rs9351963+Amrubicin and rs9351963+MMC models. Therefore, we selected the rs9351963+Amrubicin+MMC model as the best one on the basis of AIC. AUC, sensitivity, and specificity of this model were 0.744, 77.8%, and 57.6% in in the ROC curve, respectively, as shown in Figures 4A and 4B.

## Discussion

In the present study, we used 2 stages of screening: the method that is based on the concept of joint analysis. Joint analysis is more efficient than replication-based analysis [72]. The first dataset is a part of the second dataset in joint analysis (the latter includes the former). In contrast, the 2 datasets must be independent in a replication-based analysis (which we did not use here). Our 2 stages of screening derived from the joint analysis were used to increase statistical detection power. KB-SNP was performed prior to 2 stages of screening. KB-SNP reduced the number of candidate SNPs to 6,506 from 109,365. Approximately 80,000 SNPs can be extracted without knowledge-based reduction of the SNP number. Thus, statistically significant SNPs cannot be extracted from the present data. We could find the statistically significant rs9351963 in *KCNQ5* by applying the combined method to hypothesis-free genomic data.

The KCNQ/K(v)7 potassium channel family consists of 5 members of neural muscarine channel (M channel; from KCNQ1 to KCNQ5) which have a distinct expression pattern and a functional role. Although KCNQ1 is prevalently expressed in the cardiac muscle, KCNQ2, KCNQ3, KCNQ4, and KCNQ5 are expressed in neural tissue [73]–[75]. On the other hand, a recent study revealed that KCNQ4 and KCNQ5 are the most abundantly expressed KCNQ channels in smooth muscle throughout the gastrointestinal tract [76]. Furthermore, Jepps et al. opined that drugs that selectively block KCNQ4/KCNQ5 might be promising as therapeutics for the treatment of motility disorders such as constipation associated with irritable bowel syndrome [76]. In other words, drugs that selectively open KCNQ4/KCNQ5 might be effective against diarrhea. The KCNQ family gene products assemble as homomeric or heteromeric tetramers to form functional channels that mediate the M-current [77], a current that is suppressed by mAChR activation [78], [79]. Irinotecan induces adverse cholinergic effects (acetylcholinelike actions) by inhibiting AChE and binding to mAChR [37], [38], [80]. Therefore, polymorphisms of *KCNQ5* genes possibly effect incidence of diarrhea as interindividual variation in the drug response among cancer patients treated with irinotecan chemotherapy.

In the present study, only the highest grade of adverse events is recorded during the first 2 months of irinotecan treatment for each patient and each adverse effect. Therefore, incidence of grade ≥2 diarrhea possibly includes cases caused partially by enterohepatic circulation of APC and NPC, but genotype of rs9351963 in *KCNQ5* correlates with the start date of treatment with antidiarrheal agents (Spearman's rank correlation coefficient *ρ* = −0.198, *p* = 0.00995). In other words, genotype of rs9351963 may correlates with the diagnosis (or presentiment) of irinotecan induced early-onset diarrhea (diagnosis is made by trained chemotherapists).

The rs9351963 A>C polymorphism is located in an intron, which does not change the amino acid sequence of the protein and may not influence the biological function of the protein itself. Nonetheless, some intronic polymorphisms are effective markers: For example, rs2237892 in intron 15 of *KCNQ1* is associated with susceptibility to type 2 diabetes mellitus in Japanese individuals [81], and the CA simple sequence repeat in intron 1 (CA-SSR1) of the gene of epidermal growth factor receptor (*EGFR*) is associated with the clinical outcome in gefitinib-treated Japanese patients with non-small cell lung cancer [82]. Furthermore, variations related to intronic or synonymous SNPs possibly affect mRNA stability, translational kinetics, and splicing, resulting in alterations at the protein level, e.g., changes of structure or function [83]–[89]. Although rs9351963 does not have a known function, this SNP is a possible predictive factor of adverse effects of irinotecan-based chemotherapy and is possibly linked to some functional polymorphisms in *KCNQ5*. Their clinical importance needs to be further elucidated.

In the present study, we extracted rs9351963, which showed a *p* value (0.0289) obtained using a combination of 2 stages of screening and a permutation test from SNPs selected by KB-SNP. In the second dataset, the *p* value of Fisher's exact test was 3.31×10^−5^, and the *q* value was 0.173 calculated by correction of Benjamini-Hochberg method, as shown in Table 2. This value is statistically insignificant. Therefore, during the 2 stages of screening, it is statistically sufficient to extract rs9351963.

The calculation of probability of occurrence in Bernoulli trials is suitable to for estimation of validity of the repetition number in the permutation process. In this trial, occurrence probability is defined as *_n_*C*_k_*× (*p_B_*)*^k^*×(1 - *p_B_*)^(*n*–*k*)^, where *k* is the occurrence number, *n* is the repetition number, and *p_B_* represents probability. If the repetition number is 100,000 for rs9351963 (*p* = 0.02891 [2891/100000]) and the significance level of the test (α) is 0.05, the occurrence probability is _100000_C_2891_× (0.05)^2891^× (1–0.05)^(100000–2891)^  = 4.89×10^−241^. In statistics, the 99% (or 95%) confidence interval should be considered. The significance level of *α* = 0.05 does not exist in the 99% confidence interval of the *p* value for rs9351963, because the occurrence probability 4.89×10^−241^ is clearly lower than 0.01. Similarly, if the repetition number is 10,000, the occurrence probability is 3.41×10^−26^. This way, the occurrence probability is sufficiently low for 10,000 permutations. Nevertheless, we conducted 100,000 permutations to estimate *p* values more accurately for the permutation test.

Using our combined method involving KB-SNP, we identified rs9351963 as a potential predictive factor of diarrhea in cancer patients treated with irinotecan chemotherapy; however, the comprehensiveness of KB-SNP was limited. Therefore, statistical information regarding the adverse effects of cancer patients treated with irinotecan chemotherapy is shown in Table S3 for incidence of diarrhea (*p*<0.05) and in Table S4 for incidence of neutropenia (*p*<0.05). The relevant data are also provided on the website Genome Medicine Database of Japan (GeMDBJ) [90] (https://gemdbj.nibio.go.jp/). These data will be useful for replication studies or meta-analyses in the future.

In conclusion, in the present study, we applied the combined method to hypothesis-free genomic data on cancer patients treated with irinotecan chemotherapy. By means of this method, rs9351963 in *KCNQ5* was extracted as a candidate SNP related to the incidence of diarrhea. For example, the association of rs9351963 with irinotecan-related diarrhea (OR of 3.14) showed a *p* value of 3.31×10^−5^ in Fisher's exact test (allele model). Even if this *p* value were adjusted by means of the permutation test for the effects of multiple testing problems, the adjusted *p* value would still indicate statistical significance (adjusted *p* value of 0.0289<0.05). Additionally, we evaluated the performance of rs9351963 using multiple regression models. rs9351963 was clearly superior to clinical parameters (or environmental factors) and showed a sensitivity of 77.8% and specificity of 57.6% in the multiple regression model, including rs9351963. Recent studies showed that the *KCNQ4* and *KCNQ5* genes encode components of the M channel expressed in gastrointestinal smooth muscles and suggested that these genes are associated with irritable bowel syndrome and similar peristalsis diseases. These results suggest that rs9351963 may be a predictive factor of diarrhea in cancer patients treated with irinotecan chemotherapy. This SNP may also be useful for selection of chemotherapy regimens, such as irinotecan monotherapy or a combination of irinotecan chemotherapy with KCNQ5 opener. Furthermore, the result of the present analysis supports usability of our combined method.

## Supporting Information

Table S1 **Irinotecan-treated cancer patients, genetic factor, and clinical parameters for incidence of diarrhea and neutropenia.** “UGT1A1*6 or *28” is a genetic factor constructed from 2 polymorphisms (UGT1A1*6 and *28); “2” indicates *6/*6, *28/*28 or *6/*28, “1” indicates *6 or *28, and “0” indicates “other than 2 and 1.” Area: body surface area (m^2^), PS: performance status, Cr: grade of creatinine, Hg: grade of hemoglobin, Alb: grade of albumin, ALP: grade of alkaline phosphatase, and GOT: grade of glutamic oxaloacetic transaminase. Each laboratory test value (Alb, Hg, GOT, ALP, and Cr) was recorded before the irinotecan therapy. For each type of clinical tests the grade and aberrant values were defined according to the National Cancer Institute - Common Toxicity Criteria (NCI-CTC, Version 2.0). C_max_/dose: SN38 C_max_/dose [10^−3^×m^2^/L]. AUC: area under the concentration-time curve. AUC ratio: Ratio of AUC_SN38_/AUC_CPT-11_. 5-FU: 5-fluorouracil, CDDP: cisplatin, MMC: mitomycin C, VP16: etoposide. * and † indicate *p*<0.05 and 0.05≤*p*<0.10, respectively. For each concomitant drug, 0 means “not administered,” 1 indicates administered.
(XLS)

Table S2 **GO term list for transporter activity and the related functions.**
(XLS)

Table S3 **Statistical information on the chemotherapeutic response (incidence of grade ≥2 diarrhea) of irinotecan-treated cancer patients (** ***p*** **<0.05).** RS number: reference SNP identification number in dbSNP; *p* values were calculated using Fisher's exact test and *q* values were calculated using the Benjamini-Hochberg (BH) method from *p* values.
(XLS)

Table S4 **Statistical information on the chemotherapeutic response (incidence of grade ≥3 neutropenia) of irinotecan-treated cancer patients (** ***p*** **<0.05).** RS number: reference SNP identification number in dbSNP; *p* values were calculated using Fisher's exact test and *q* values were calculated using the Benjamini-Hochberg (BH) method from *p* values.
(XLS)
